# Complications of transcatheter paravalvular leak device closure of mitral valve: An updated review of the literature and a rare case presentation

**DOI:** 10.1002/clc.24272

**Published:** 2024-05-14

**Authors:** Yaser Jenab, Saeed Nourian, Negin Sadat Hosseini Mohammadi, Reza Mohseni‐Badalabadi, Kaveh Hosseini, Sarina Zakavi, Ismail Ates

**Affiliations:** ^1^ Tehran Heart Center, Cardiovascular Diseases Research Institute Tehran University of Medical Sciences Tehran Iran; ^2^ Firoozgar Clinical Research and Development Center Iran University of Medical Sciences Tehran Iran; ^3^ Cardiac Primary Prevention Research Center, Cardiovascular Diseases Research Institute Tehran University of Medical Science Tehran Iran; ^4^ Students' Scientific Research Center (SSRC) Tehran University of Medical Science Tehran Iran; ^5^ Faculty of Healthscience Bahcesehir University Istanbul Turkey

**Keywords:** heart failure, hemolytic anemia, paravalvular leak, percutaneous closure, valvular prosthesis

## Abstract

Paravalvular leak (PVL) is an uncommon complication of prosthetic valve implantation, which can lead to infective endocarditis, heart failure, and hemolytic anemia. Surgical reintervention of PVLs is associated with high mortality rates. Transcatheter PVL closure (TPVLc) has emerged as an alternative to surgical reoperation. This method provides a high success rate with a low rate of complications. This article reviews the pathogenesis, clinical manifestation, diagnosis, and management of PVL and complications following TPVLc. Besides, we presented a case of a patient with severe PVL following mitral valve replacement, who experienced complete heart block (CHB) during TPVLc. The first TPVLc procedure failed in our patient due to possible AV‐node insult during catheterization. After 1 week of persistent CHB, a permanent pacemaker was implanted. The defect was successfully passed using the previous attempt. Considering the advantages of TPVLc, procedure failure should be regarded as a concern. TPVLc should be performed by experienced medical teams in carefully selected patients.

## Graphical abstract

We offer updated insights into the pathogenesis, clinical manifestations, diagnosis, and management of paravalvular leak (PVL), as well as the complications ensuing from transcatheter PVL closure (TPVLc). Additionally, we present a case study involving a patient afflicted by severe PVL subsequent to mitral valve replacement, who encountered a significant complication in the form of complete heart block during TPVLc.

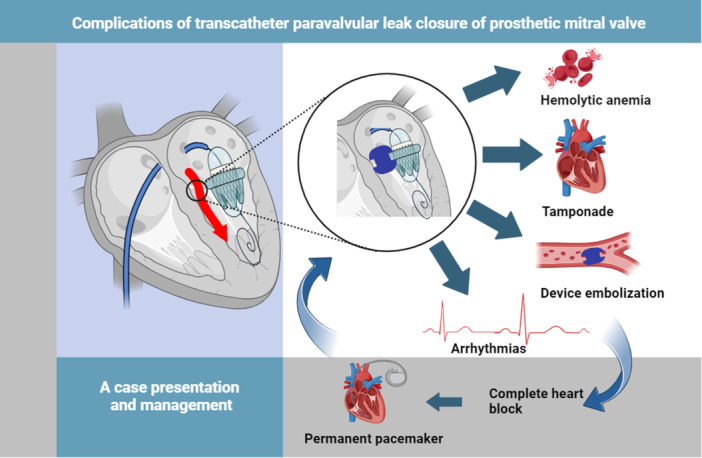

**Abbreviations**
3D‐TEE: three‐dimensional trans‐esophageal echocardiography
CF: cinefluoroscopy
CHB: complete heart block
CT: computed tomography
ICE: intracardiac echocardiography
MR: magnetic resonance
MVR: mitral valve replacement
NT‐proBNP: N‐terminal pro‐B‐type natriuretic peptides
PPM: permanent pacemaker
PVL: paravalvular leak
TPVLc: transcatheter PVL closure
TTE: transthoracic echocardiography

## 1 INTRODUCTION

Paravalvular leak (PVL) is a life‐threatening complication of prosthetic valve replacement, which affects approximately 7%−17% of patients with mitral valve prostheses.^1^ This relatively uncommon yet grave complication primarily ensues from factors such as annular calcification, suboptimal suture lines attributable to technical errors, suture failure, annular disruption, and endocarditis.^2^ Patients with severe PVL are at risk of developing serious clinical consequences, including progressive heart failure and hemolytic anemia.^3^ Although the recommended intervention is to redo surgery, the transcatheter PVL closure (TPVLc) method has garnered considerable favor within contemporary clinical practice due to being safe, effective, and minimally invasive nature. While this method has been reported to achieve a high success rate, it's associated with several complications encompassing stroke, complete heart block (CHB), cardiac perforation and tamponade, and death.^2^, ^3^ Herein, we undertake a literature survey, delving into the clinical implications, diagnosis, and therapeutic modalities pertinent to PVLs. Moreover, we scrutinize the landscape of complications and outcomes associated with TPVLc. To further illuminate the complexity of this issue, we present a case study featuring a patient afflicted by severe PVL following mitral valve replacement (MVR) and concomitant pulmonary hypertension, who encountered a rarely reported complication: CHB during TPVLc.

## 2 CASE PRESENTATION

**Figure 1:**
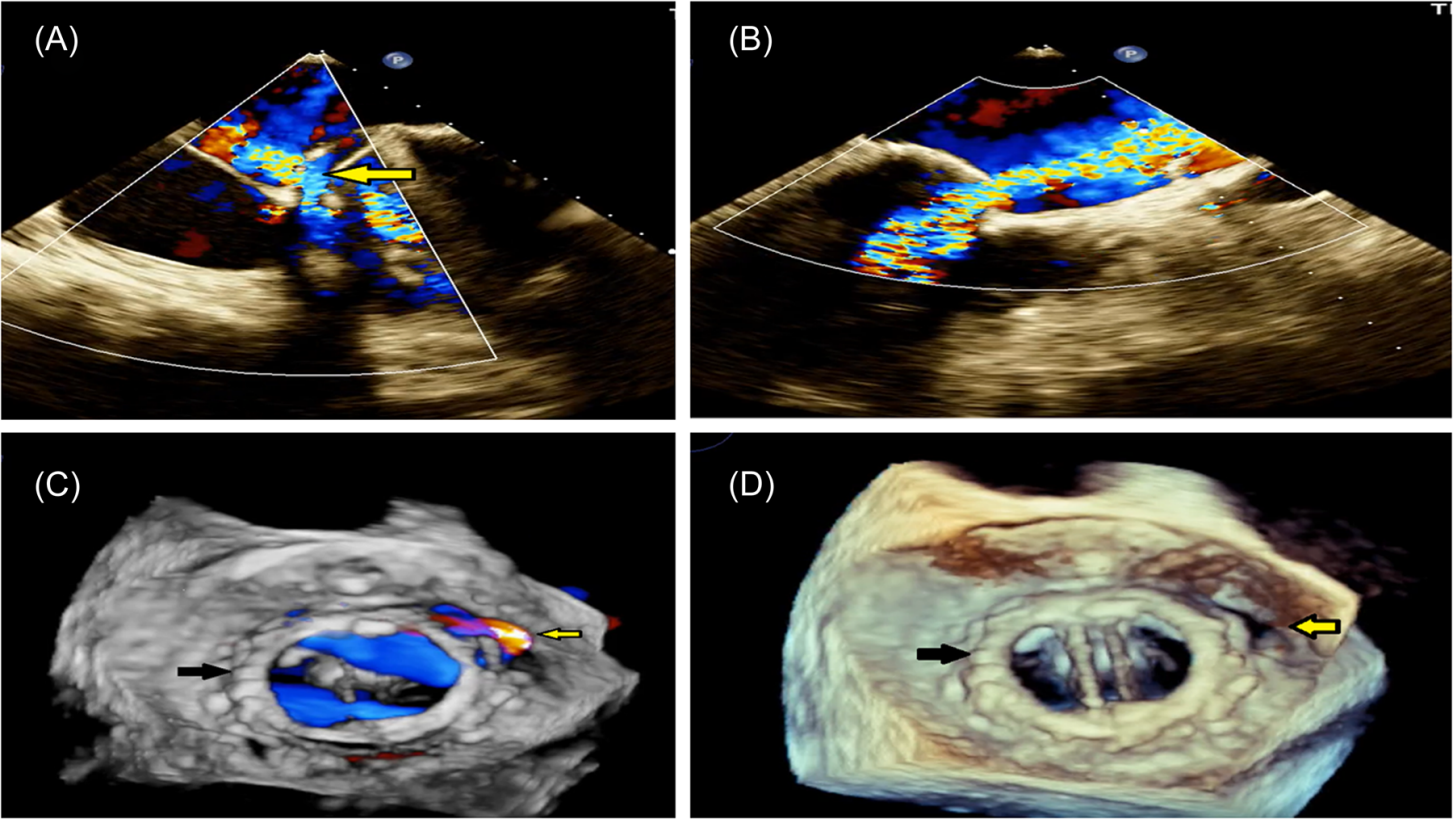
(A) TEE 4 chamber view. Dehiscence of the medial side of MV sewing ring. Severe paravalvular leakage (yellow arrow). (B) TEE parasternal view. Dehiscence of the anterior side of MV sewing ring. Severe paravalvular leakage. (C) 3D TEE color. Surgical view of prosthetic MV (black arrow) with paravalvular leakage (yellow arrow). (D) Surgical view of prosthetic MV (black arrow) with a paravalvular defect in the anteromedial side of MV sewing ring (yellow arrow). TEE, transesophageal echocardiography.

**Figure 2:**
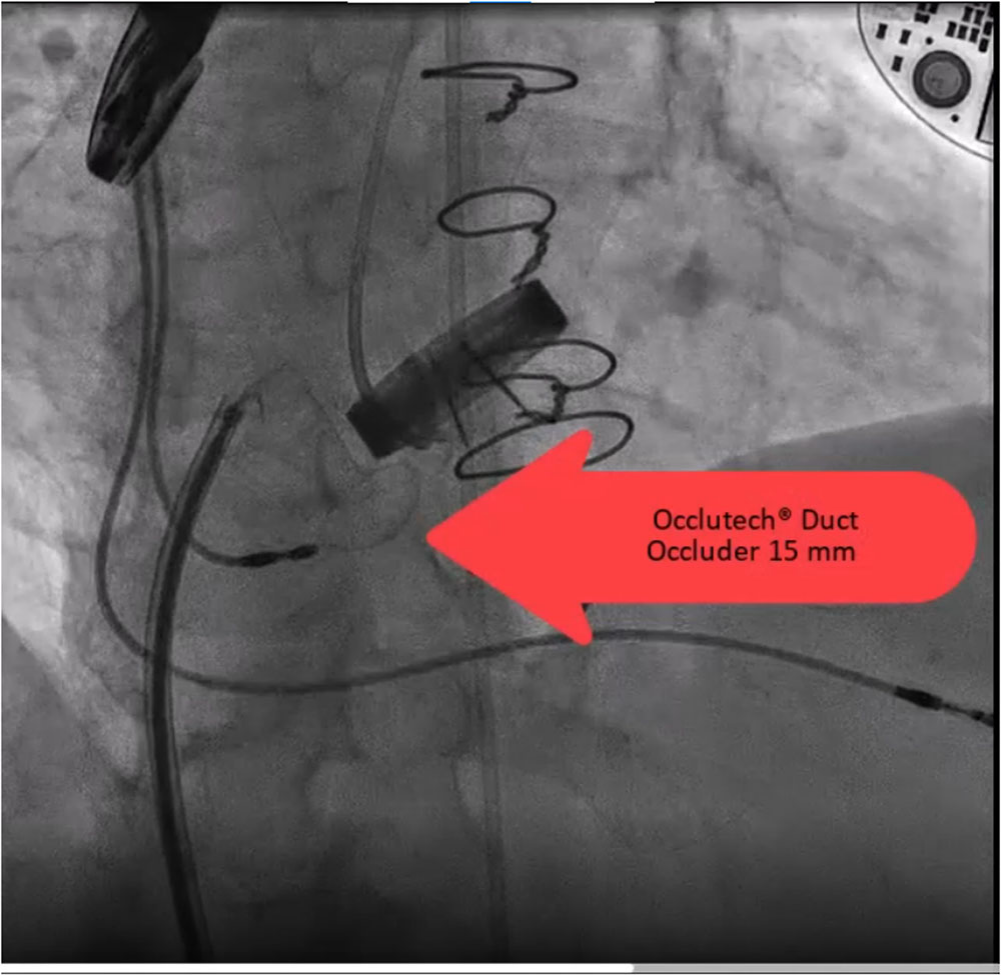
Finally PDA occluder size 15 was implanted with no complication. PDA, patent ductus arteriosus.

The patient is a 61‐year‐old female with a past medical history of exertional dyspnea (first documented 3 years before her current admission) and 24 mm St. Jude Medical mechanical mitral valve prosthesis (approximately 12 years before her present admission). The patient was referred to Tehran Heart Center due to worsening exertional dyspnea (NYHA class IV) (Table 1). The patient's blood pressure was 127/66 mmHg, heart rate was 85 bpm, and oxygen saturation was 95% in room air. On physical examination, a holosystolic murmur at the left sternal border was heard with a mechanical heart sound. Laboratory investigations unveiled a hemoglobin level of 12.5 mg/dL, LDH (lactate dehydrogenase) levels at 345 U/L, direct bilirubin at 0.7 mg/dL, and a total bilirubin level of 1.3 U/L, all indicative of the absence of hemolytic anemia at the time of admission. The electrocardiography (ECG) showed preexisting atrial fibrillation combined with a left bundle branch block. Coronary CT angiography revealed mild coronary artery disease. Three‐dimensional transesophageal echocardiography (3D‐TEE) showcased a left ventricular ejection fraction of approximately 50% with severe enlargement, although the mechanic prosthetic mitral valve was functioning normally, Further scrutiny using 3D‐TEE elucidated the presence of severe PVL localized at the anteromedial part of the ring (1−3 O'clock surgical view) on a complex, irregularly shaped defect measuring 11 mm × 6 mm (Figure 1). The right ventricle had moderate dysfunction with systolic pulmonary artery pressure of 68 mmHg. Considering the patient's history of previous surgery and the presence of severe pulmonary hypertension, the decision was made to proceed with percutaneous PVL closure. We decided to perform the procedure using the antegrade transseptal transcatheter technique. Under general anesthesia, through the femoral vein, a transseptal puncture was made to access the left atrium. Subsequently, through intravenous access, via the left atrium, after inter‐atrial septostomy and introducer implanted (Steerable Agilis NxT) in the left atrium, over the Amplatz Super Stiff 0.035 guidewire (Boston Scientific) delivery catheter introduced and an 15 mm Occlutech PLD Occluder (Occlutech) was tried to deploy at the PVL site. Unexpectedly the cardiac rhythm changed to the CHB with consequent hemodynamic compromise. The delivery sheath was then withdrawn back into the left atrium, and a temporary pacemaker was rapidly placed in the right atrium. Further attempts to cross the defect were unsuccessful. After 1 week of the persistent CHB, a dual chamber permanent pacemaker (PPM) (DDDR St Jude's Medical‐Abbott) was implanted (Figure 2).

**Table 1:** Summary of the events.

Admission day	A 61‐year‐old female patient was referred to our center due to worsening exertional dyspnea (NYHA class IV)
Assessments following admission	(1) The ECG showed preexisting AF combined with a left bundle branch block. (2) Coronary CTA revealed mild CAD. (3) 3D‐TEE showed a left ventricular ejection fraction of approximately 50% with severe enlargement. (4) 3D‐TEE elucidated the presence of severe PVL localized at the anteromedial part of the ring, moderate right ventricle dysfunction, and systolic pulmonary artery pressure of 68 mmHg
First PVLC attempt following initial diagnosis	The cardiac rhythm changed to the CHB before delivery of the device, and attempts to recross the defect were unsuccessful. A temporary pacemaker was placed in the RA
1 week after the first intervention	A dual chamber PPM was implanted due to persistent CHB
2nd month following the intervention	The patient was rescheduled for a second TPVLc attempt. The defect was passed using the antegrade transseptal approach with real‐time guidance provided by 3D‐TEE
10‐days after the second TPVLc intervention	The patient was discharged in a satisfactory general condition

Abbreviations: 3D‐TEE, 3D transesophageal echocardiography; AF, atrial fibrillation; CAD, coronary artery disease; CHB, complete heart block; CTA, computed tomography angiography; ECG, electrocardiography; NYHA, The New York Heart Association classification; PPM, permanent pacemaker; PVL, paravalvular leak; RA, right atrium; TPVLc, transcatheter paravalvular leak device closure.

**Figure 3:**
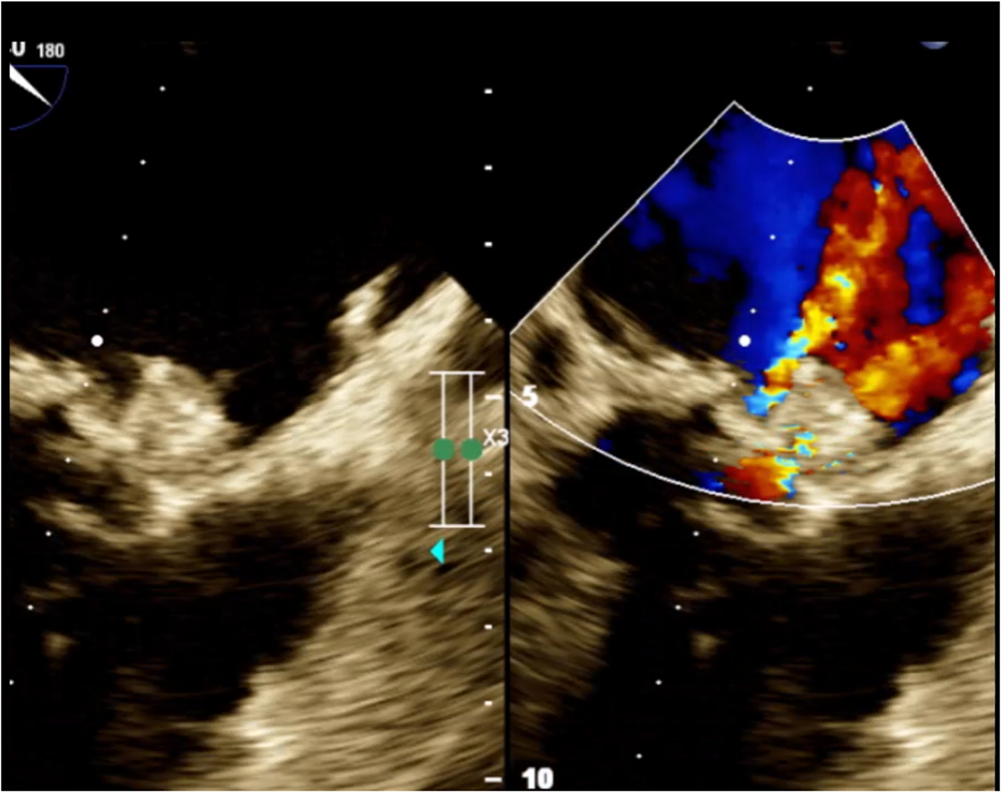
TEE showed mild residual paravalvular leakage. TEE, transesophageal echocardiography.

Over the course of 2 months following these interventions, the patient's persistent paced rhythm and dyspnea raise the concern for worsening PVL. Accordingly, the patient was readmitted and rescheduled for a subsequent transcatheter closure. The defect was passed easily using the previously attempted method (antegrade transseptal) with real‐time guidance provided by 3D‐TEE. A 15 mm Occlutech PDA Occluder was deployed successfully with mild residual leakage and had no interference with mitral mechanical valve leaflets (Figure 3). During the procedure, PPM was working properly with no evidence of heart block or hemodynamic instability.

Following the second procedure, the patient's dyspnea improved immediately to NYHA class Ⅱ. However, she experienced two episodes of hematuria within a few hours and 5 days' postprocedure, each episode lasting approximately 24 and 48 h, respectively. Postprocedural laboratory studies were in favor of hemolytic anemia (hemoglobin: 8.5 mg/dL, LDH: 1759 IU/L, total bilirubin: 2.7 mg/dL, and direct bilirubin: 1.36 mg/dL). One unit of packed cells was transfused to address the hemolytic anemia. Follow‐up ECG revealed mild mitral paravalvular leakage with preserved prosthetic leaflet function. Throughout a 10‐day follow‐up period, the laboratory turned to normal values, and the patient was discharged in satisfactory general condition.

## 3 PATHOGENESIS AND CLINICAL MANIFESTATIONS OF PVLS

PVLs are uncommon but serious complications of valve replacements which more tend to occur in MVRs (7%–17%), compared to aortic valve replacements (5%–10%).^4^, ^5^ They are characterized by a regurgitant blood flow through a gap between adjacent tissue of the native valve annulus and a prosthetic ring.^6^ Numerous predisposing factors have been implicated in the development of PVLs, including annular calcification, tissue friability, infectious endocarditis, increased atrial size, corticosteroid therapy, old age, renal insufficiency, mechanical implants, and continuous sutures.^7^, ^8^, ^9^ Furthermore, it is noteworthy that specific segments of the mitral valve annulus, particularly the anterolateral and posteromedial regions, exhibit heightened susceptibility to PVL formation. This susceptibility can be attributed to variations in collagen fiber distribution, longer annular dimensions in the posterior segment, and alterations in the dynamics of the mitral valve annulus subsequent to MVR, collectively leading to increased mechanical stress on these specific regions.^10^, ^11^

Although the majority of PVLs are clinically trivial or mild and have a benign course, clinically significant PVLs are encountered in approximately 1%–5% of implanted valves.^12^ Large PVLs may result in clinically significant manifestations, including heart failure, hemolytic anemia, and infectious endocarditis.^13^, ^14^, ^15^ The clinical symptoms of mitral PVLs are predominantly dependent on the size of the leak. Smaller PVLs primarily result in hemolysis, whereas large PVLs are more likely to cause symptoms of heart failure.^16^, ^17^ However, a previous study demonstrated that the degree of hemolysis in mitral PVL was not related to PVL size. Although it established a significant association between the hemodynamic index of pulmonary hypertension and PVL size.^18^ For cases involving small PVLs devoid of substantial hemolysis, the preferred, the method of choice is the conservative treatment, encompassing regular monitoring of laboratory parameters and ultrasound evaluation. Conversely, in instances where the PVL is hemodynamically significant, the method of choice is cardiac surgery.^19^ Consequently, further evidence to support clinical decisions for PVL closure based on size is needed. An important part of planning the percutaneous PVL closure is comprehensively understanding the shape and size of the defect. Large mitral PVLs often manifest with a holosystolic murmur at the left sternal border or in the midaxillary line.^20^ Upon Laboratory analysis, blood tests can be helpful to suspect hemolysis (hemoglobin ≤10 g/dL, LDH ≥600 mg/dL, and haptoglobin ≤10 mg/dL).^13^ Furthermore, renal function may be altered due to an increase of N‐terminal pro‐B type natriuretic peptides (NT‐proBNP) following heart failure. Furthermore, to investigate the possibility of infective endocarditis, blood cultures may be warranted.^21^, ^22^

## 4 DIAGNOSTIC METHODS

Multiple imaging modalities are available for the diagnosis and comprehensive assessment of PVLs, including transthoracic echocardiography (TTE), transesophageal echocardiography (TEE), intracardiac echocardiography (ICE), computed tomography (CT), magnetic resonance (MR), and cinefluoroscopy (CF). TTE is an essential diagnostic test of choice for initial patient work‐up for all patients with suspected PVL. TTE exhibits a sensitivity and specificity of 57% and 63%, respectively, for identifying valvular abnormalities.^23^ Although TTE can provide helpful assessment regarding valvular gradients, the utility of TTE may be constrained by the presence of acoustic shadows and artifacts, potentially impeding accurate estimation of PVL severity. Additionally, prosthetic‐related acoustic shadowing may lead to the absence of color doppler signal which makes it difficult to delineate valvular versus paravalvular regurgitation.^24^, ^25^ Therefore, TEE emerges as the preferred modality when there is a high suspicion for structural abnormalities and adds value to determine the degree of regurgitation and valve dysfunction.^26^ Two‐dimensional TEE (2D) provides a sensitive assessment regarding PVL identification; however, three‐dimensional TEE (3D) has several advantages over 2D‐TEE in the assessment of the PVLs, especially in complex defects.^27^ Indeed, 3D‐TEE is the gold standard for PVL evaluation and provides essential guidance for percutaneous PVL closure procedures.^28^ ICE, although less frequently employed due to the potential for acoustic shadowing, retains utility as it can be performed without the need for general anesthesia.^29^ CF modality plays a crucial role during catheterization providing complementary data to echocardiographic views.^30^ In the realm of preprocedure assessment for transcatheter valve interventions, the significance of CT and MRI has been steadily on the rise, providing a complementary dimension to echocardiography. Despite recent improvements in both hardware and software, TTE measurement of PVLs largely remains qualitative. Recent studies highlight a pronounced tendency for TTE to underestimate PVLs when compared to CMR imaging.^31^, ^32^ In contrast to echocardiography, CMR stands out for its ability to directly quantify PVLs with exceptional accuracy and reproducibility, employing the technique of phase‐contrast velocity mapping. Phase‐contrast velocity mapping is performed in the short‐axis plane just distal to the prosthetic valve, facilitating subsequent quantification of regurgitant volume and regurgitant function.^32^ Notably, CMR is not affected by the location, number, or nature of regurgitant jets or thoracic structural patient factors. Therefore, emerges as an optimal technique for assessing the severity of PVLs, particularly in the presence of multiple and eccentric leaks or acoustic shadows that can compromise the accuracy of echocardiography.^33^, ^34^

## 5 TREATMENT

Symptomatic or severe PVLs entail to be addressed by either a surgical reintervention or TPVLc.^35^ Surgery is recommended in patients present with severe PVL, refractory heart failure, failed conservative medical management, or in cases where TPVLc is contraindicated.^36^ Active endocarditis or vegetation and regurgitation involving more than one‐third of the circumference of the prosthetic annulus are among the contraindications for TPVLc.^37^ Although surgical reintervention has been considered the treatment of choice for symptomatic patients with PVLs, it carries considerable risks, including 8.8%−11.5% of 30‐day in‐hospital mortality.^38^, ^39^ TPVLc is a less invasive alternative for surgical reoperation of PVLs, particularly in patients with high or prohibit risk of surgery.^40^ TPVLc is usually adopted as the first‐line strategic approach in many experienced centers because, essentially, surgical reintervention procedures carry moderate to high risk. In rare instances, reconsideration of surgical repair may be contemplated following TPVLc failure.^37^ It is essential to select the appropriate TPVLc techniques when managing PVLs. A variety of percutaneous technique including antegrade approach, retrograde approach and transapical access are utilized for PVL treatment according to the type of valve and leak positioning.^41^ There is limited data regarding the optional approach for PVLs and various approaches are adopted in experienced centers. The antegrade transseptal approach is frequently favored for managing mitral PVLs. The retrograde transapical approach is preferred when the transeptal approach is not achievable. Additionally, a retrograde femoral artery approach with venous access is commonly employed for addressing aortic valve leaks.^42^

## 6 COMPLICATIONS AND OUTCOMES

TPVLc has emerged as a safe alternative for surgical reintervention with a high procedural success rate of about 77%−91% and favorable long‐term survival of the patients.^43^ The achievement of procedural success is contingent upon several independent predictors, including the severity of regurgitation, the size of the leak, the choice of device utilized, and the expertise of the operator.^44^, ^45^ Large size, irregular, and slope tunnel‐shaped and multiple PVLs have been associated with reduced success rates.^46^

Despite the promising outcomes and high success rate, TPVLc is a challenging and demanding procedure. Access site complications of TPVLc are rare due to the transvenous approach; nevertheless, several complications are associated with the transvenous approach, including a free wall or organ perforation and systemic embolization of thrombus, hemothorax, tamponade, vascular laceration, and pneumothorax.^47^, ^48^ Furthermore, risk of leaflet entrapment by the device, leaflet erosion by large devices, sustained radial forces on the surrounding tissue and tear of tissues are among the device‐related complications following TPVLc.^49^, ^50^

Mechanical valve obstruction, worsening of hemolysis, thromboembolic complications, and life‐threatening arrhythmias are the most serious TPVLc‐related complications.^17^, ^51^ The friction of erythrocytes with the rough surface of the metal surface of the closure device increases the potential for new or worsening intravascular mechanical hemolysis.^52^ However, the risk of hemolysis is reduced by using smaller devices enables to achieve a higher sealing with less residual leakage.

Device embolization count as another complication of TPVLc which rarely occur when the closure device is inadvertently released and cannot be retrieve.^13^ The release mechanism usually can be controlled by the operator and the device is securely located inside the leak tunnel. Besides, the embolized device can usually be retrieved percutaneously; therefore, the risk of device embolization is relatively low.^43^

Cruz‐González et al. have reported one‐third‐degree AV block requiring temporary pacemaker implantation following 128 consecutive percutaneous mitral PVL closure procedures performed in 96 patients.^2^ As the AV node is located at the junction of inter‐atrial and interventricular septum, TPVLc intervention at this site can be complicated by CHB, however, the exact mechanism of injury remains elusive.

Considering the advantages of TPVLc, procedure failure should be regarded as a concern, although complications have not occurred, as the patient will require reoperation with a higher morbidity and mortality. The first TPVLc procedure failed in our patient due to possible AV‐node insult during catheterization. After 1 week of the persistent CHB, a PPM was implanted. Although it has been shown that repeated attempts at TPVLc in surgery‐ineligible patients decrease the success rate,^53^ the defect was successfully passed using the previous attempt.

## 7 CONCLUSIONS

Accumulating clinical data implies the evidence for the successful use of TPVLc intervention. Although limited head‐to‐head data is comparing the two treatment modalities, TPVLc offers several potential advantages over surgical reintervention, most notably a higher technical success rate and lower risk of complications. Early experience has shown promising results; however, the optimization of long‐term outcomes necessitates further exploration through registry‐based studies. Additionally, it is worth acknowledging that the management of complications arising post‐TPVLc remains an area with limited available data. Further, the predictors for developing post‐TPVLc complications are still controversial, and a more thorough evaluation is warranted.

## CONFLICT OF INTEREST STATEMENT

The authors declare no conflict of interest.

## Supporting information

Supporting information.

Supporting information.

Supporting information.

## Data Availability

Data sharing is not applicable to this article as no data sets were generated or analyzed during the current study.
