# Supplementary material for: Complications of transcatheter paravalvular leak device closure of mitral valve: An updated review of the literature and a rare case presentation
Source: Clin Cardiol. 2024 May 14;47(5):e24272. doi: 10.1002/clc.24272 (PMC11092304; doi:10.1002/clc.24272)
Supplement: Supplementary file 3 — Supporting information. [file CLC-47-e24272-s003.docx]

**Dear Editor in Chief of Clinical Cardiology;**

The authors of the manuscript entitled ***“Complications of trans-catheter paravalvular leak device closure of mitral valve: An update review of literature and a rare case presentation”*** declare that they are employees of Tehran University of Medical Sciences, Iran University of Medical Sciences, and Bahcesehir University that are governmental agencies and their primary functions are:

1-     Medical education

2-     Medical research

3-     Medical treatment

4-     Primary health care service

Also, we declare that the mentioned manuscript is being submitted by the authors and that **“none of them”** are official representatives or on behalf of the government.

Best regards,

**Reza Mohseni-Badalabadi, MD (Corresponding Author)**

Tehran Heart Center, Kargar St. Jalal al-Ahmad Cross, Tehran, 1411713138, Iran.

Email: [mohsenihr@yahoo.com](mailto:mohsenihr@yahoo.com)

Tel.: +98-21-88029600; Fax: +98-21-88029614.

**Yaser Jenab, MD**

([yasjenab@gmail.com](mailto:yasjenab@gmail.com)) (ORCID: 0000-0002-8291-8637)

Tehran Heart Center, Cardiovascular Diseases Research Institute, Tehran University of Medical Sciences, Tehran, Iran

**Saeed Nourian**

([S.nourian@gmail.com](mailto:S.nourian@gmail.com))

Firoozgar Clinical Research and Development Center, Iran University of Medical Sciences, Tehran, Iran

Negin Sadat Hosseini Mohammadi

([neghhm98@gmail.com](mailto:neghhm98@gmail.com)) (ORCID:0000-0002-9477-6584)

Tehran Heart Center, Cardiovascular Diseases Research Institute, Tehran University of Medical Sciences, Tehran, Iran

Students’ Scientific Research Center (SSRC), Tehran University of Medical Science, Tehran, Iran

Kaveh Hosseini, MD

([Kveh_hosseini130@yahoo.com](mailto:Kveh_hosseini130@yahoo.com))

Tehran Heart Center, Cardiovascular Diseases Research Institute, Tehran University of Medical Sciences, Tehran, Iran

Cardiac Primary Prevention Research Center, Cardiovascular Diseases Research Institute, Tehran University of Medical Science, Tehran, Iran

Sarina Zakavi, MD

([sarinazakavi@yahoo.com](mailto:sarinazakavi@yahoo.com))

Tehran Heart Center, Cardiovascular Diseases Research Institute, Tehran University of Medical Sciences, Tehran, Iran

Students’ Scientific Research Center (SSRC), Tehran University of Medical Science, Tehran, Iran

Ismail Ates, MD

([drismailates@gamil.com](mailto:drismailates@gamil.com))

Bahcesehir University, Faculty of Healthscience, Istanbul, Turkey

**Hamidreza Soleimani,** **MD**

Tehran Heart Center, Cardiovascular Diseases Research Institute, Tehran University of Medical Sciences, Tehran, Iran

**Behrad Saeedian,** **MD**

School of Medicine, Tehran University of Medical Sciences, Tehran, Iran

**Yeganeh Pasebani,** **MD**

Rajaie Cardiovascular Medical and Research Center, Iran University of Medical Sciences, Tehran, Iran

**Nastaran Babajani,** **MD**

School of Medicine, Tehran University of Medical Sciences, Tehran, Iran

**Amirreza Pashapour Yeganeh,** **MD**

School of Medicine, Tehran University of Medical Sciences, Tehran, Iran

**Pegah Bahirai,** **MD**

Shahid Beheshti University of Medical Science, Tehran, Iran

**Hossein Navid,** **MD**

Tehran Heart Center, Cardiovascular Diseases Research Institute, Tehran University of Medical Sciences, Tehran, Iran

**Ahmad Amin,** **MD**

Rajaie Cardiovascular Medical and Research Center, Iran University of Medical Sciences, Tehran, Iran
